# Social-ecological factors related to preventive behaviors during the COVID-19 pandemic in South Korea

**DOI:** 10.1371/journal.pone.0266264

**Published:** 2022-03-31

**Authors:** Sou Hyun Jang

**Affiliations:** Department of Sociology, Korea University, Seongbuk-gu, Seoul, South Korea; Post Graduate Institute of Medical Education and Research (PGIMER), INDIA

## Abstract

Most studies on COVID-19 preventive behaviors have focused on single-level factors such as national policy, community social capital, or individuals’ sociodemographic characteristics. Through a social-ecological model, this study attempts to comprehensively examine the multilevel factors associated with COVID-19 preventive practices in South Korea. Accordingly, a web survey involving 1,500 participants was conducted in December 2020. An ordinary least squares (OLS) regression was used to examine the multilevel factors (individual, interpersonal, community, and policy levels) related to COVID-19 preventive measures, which are based on wearing a mask, washing hands, covering the mouth when coughing or sneezing, and social distancing. When factors at each level were investigated, higher scores of COVID-19 fear and correct knowledge at the individual level, COVID-19 information share at the interpersonal level, and better evaluation of the national government policies in regard to COVID-19 at the policy level were positively associated with COVID-19 preventive behaviors. Community-level factors—neighborhood perception and community participation—were negatively significantly related to COVID-19 preventive behaviors. Additionally, older age, being female, and having a graduate-level education were positively related to better preventive behaviors. The findings of the current study suggest that multilevel efforts are needed to promote preventive behaviors. Specifically, more effort to alleviate COVID-19-related fear and disseminate correct knowledge among Korean citizens is needed as the individual-level characteristics explained the preventive behaviors more than the factors at upper levels.

## Introduction

At the beginning of the coronavirus disease 2019 (COVID-19) outbreak, each nation’s response was different, ranging from herd immunity to lockdowns [1, 2]. South Korea (hereinafter, Korea) experienced an early outbreak of COVID-19, but the Korean government flattened the curve [3] by rapidly responding to COVID-19 with strong national leadership [4] as it proactively and efficiently conducted COVID-19 testing, which included the introduction of drive-through and walk-through COVID-19 testing services [5]. The Korean government also actively utilized information technology (IT) to disseminate COVID-19 information via web posts, text alerts, and contact traces [3, 5] and to fight against misinformation [6].

Along with the national-level factors, individual-level preventive behaviors, such as following social distancing and wearing a mask, could also contribute to the prevention of the spread of COVID-19 [1, 7, 8]. For example, the “critical support from Korean citizens” also contributed to the COVID-19 situation in Korea in addition to national-level policies [3]. Previous studies have found that several demographic characteristics at the individual level, such as age [9–11], gender [10–12], and political orientation [13], are associated with the practice of COVID-19 preventive measures in various countries, including the United States, the Kingdom of Saudi Arabia, Malaysia, and Bangladesh. In general, people who are older, women, and those with left-leaning political orientation tend to practice COVID-19 preventive measures better than the younger population, men, and right-leaning groups [13, 14]. In addition to the demographic characteristics, individuals’ better knowledge [8, 10], positive attitude toward COVID-19 [11], and fear of COVID-19 [12] are positively associated with their preventive behaviors. According to comparative studies that examined risk perception among individuals across 10 countries [8], risk perception is related to preventive behaviors. Additionally, being female, having a liberal political ideology, more personal knowledge, direct experience with COVID-19, and trust in medical professionals were positively related to risk perceptions, while trust in government was negatively related to it in South Korea.

In addition to national-level and individual-level factors, COVID-19 preventive behaviors were also found to be based on the factors in between these levels, such as civic capital [15] or social capital [16–18], while civic capital is defined as the “set of values and beliefs that help a group overcome the free-rider problem in the pursuit of socially valuable activities” [15, 19] and social capital refers to the “civic norms and social networks that facilitate collective actions and foster cooperation and trust within a community” [16, 20, 21]. Previous studies have found that a higher degree of civic or social capital positively impacts an individual’s or community’s social distancing, which is one of the recommended preventive measures by the World Health Organization (WHO) [22] and the Centers for Disease Control and Prevention [23]. Communities with a higher degree of social capital are more likely to be tested for COVID-19 [24] and practice social distancing [17] than communities with a lower degree of social capital because social capital has both direct (through quick dissemination of correct COVID-19 information) and indirect (through changes in individuals’ awareness and evaluations of preventive behaviors) pathways for impacting social distancing. Although pioneering studies have focused on either social distancing [15–17] or covering one’s face with a mask [13, 25] rather than considering inclusive preventive measures, a more thorough examination of the impact of community-level factors on other preventive behaviors is needed, including wearing a mask and washing hands, which should become a daily practice.

### Theoretical framework: Social-ecological model

Most studies have focused on single-level factors, including individual, community, or national, which are related to preventive behaviors. While the factors between national and individual levels are understudied, these could be important, and the current study tries to fill the gap in the literature in this regard. The social-ecological model indicates that there are different multilevel factors that are interrelated and can impact health behaviors [26–29]. For example, the model suggests that factors at the individual level (e.g., age, gender, race/ethnicity, perception, and attitude) and those beyond, such as family relationships at the interpersonal level, neighborhood support at the community level, and national policy at the policy level, should be considered when examining health behavior.

Previously, during the pre-COVID-19 period, the social-ecological model was applied to develop, implement, and evaluate health promotion interventions [30, 31]. Nonetheless, during the pandemic, a few studies have applied the social-ecological model to examine COVID-19 vaccine trust [32], mental health outcomes among healthcare workers [33], wearing a face covering [13], risk perception of COVID-19 [8], and adherence to COVID-19 related advice [34]. Accordingly, these studies have found that different multilevel factors contribute to various outcomes. Thus, I hypothesize that different factors at the individual, interpersonal, community, and policy levels have contributed to preventive behaviors during the COVID-19 pandemic in Korea.

### The current study

Applying the social-ecological model, this study aims to examine the multilevel factors associated with different preventive behaviors during the COVID-19 pandemic in Korea. The study population included Korean citizens aged 19–69 years who were recruited online in December 2020. In addition to the well-examined social distancing, it includes other preventive behaviors, which are based on wearing a mask, washing one’s hands after going out, washing one’s hands before meals, and covering one’s mouth when coughing and sneezing. In this paper, I ask two critical questions: a) How well do Koreans practice COVID-19 preventive measures? and b) What multilevel factors determine preventive behaviors? As discussed in previous studies [1, 7, 8], understanding and promoting an individual’s preventive behaviors is important because it could fundamentally contribute to stopping the pandemic. The findings of this study will contribute to the growing, yet limited, literature on COVID-19 with suggestions for future studies and public health implications to tailor various policies at different levels.

## Methods

### Data

The current study used a quantitative method. An online survey of 1,500 Koreans aged 19–69 years was conducted in December 2020. The survey participants were recruited by Research & Research, an online research company with a commercialized research panel in South Korea. Since it was not feasible for me to obtain a list of all Koreans, random sampling was not possible. Instead, to increase the representativeness of the population, quota sampling was carried out based on age, sex, and area of residence. Participants who were not adults (i.e., ones younger than 19 years) and who did not indicate that they were of Korean ethnicity were excluded. Everyone who agreed to participate in the survey received a website link with the survey questions, and their answers were handled and saved in Microsoft Excel. It took approximately 10–30 minutes to complete the survey. This study was approved by the Institutional Review Board (IRB #2020-11-008) with which I am affiliated. Written informed consent was obtained from all the participants. Interested researchers might replicate the current study by requesting and obtaining data from the Presidential Commission on Policy Planning of South Korea and following the protocol.

### Measures

The dependent variable was individuals’ COVID-19 preventive practices; it was assessed using five items scored on an 11-point scale: 1) wearing a mask, 2) washing hands after going out, 3) washing hands before meals, 4) covering the mouth when coughing and sneezing, and 5) social distancing. Each response ranged from “do not practice at all” (0 points) to “always practice” (10 points). The mean was used in the analysis, and Cronbach’s alpha (α) was 0.8903.

#### Individual level

**Fig 1:**
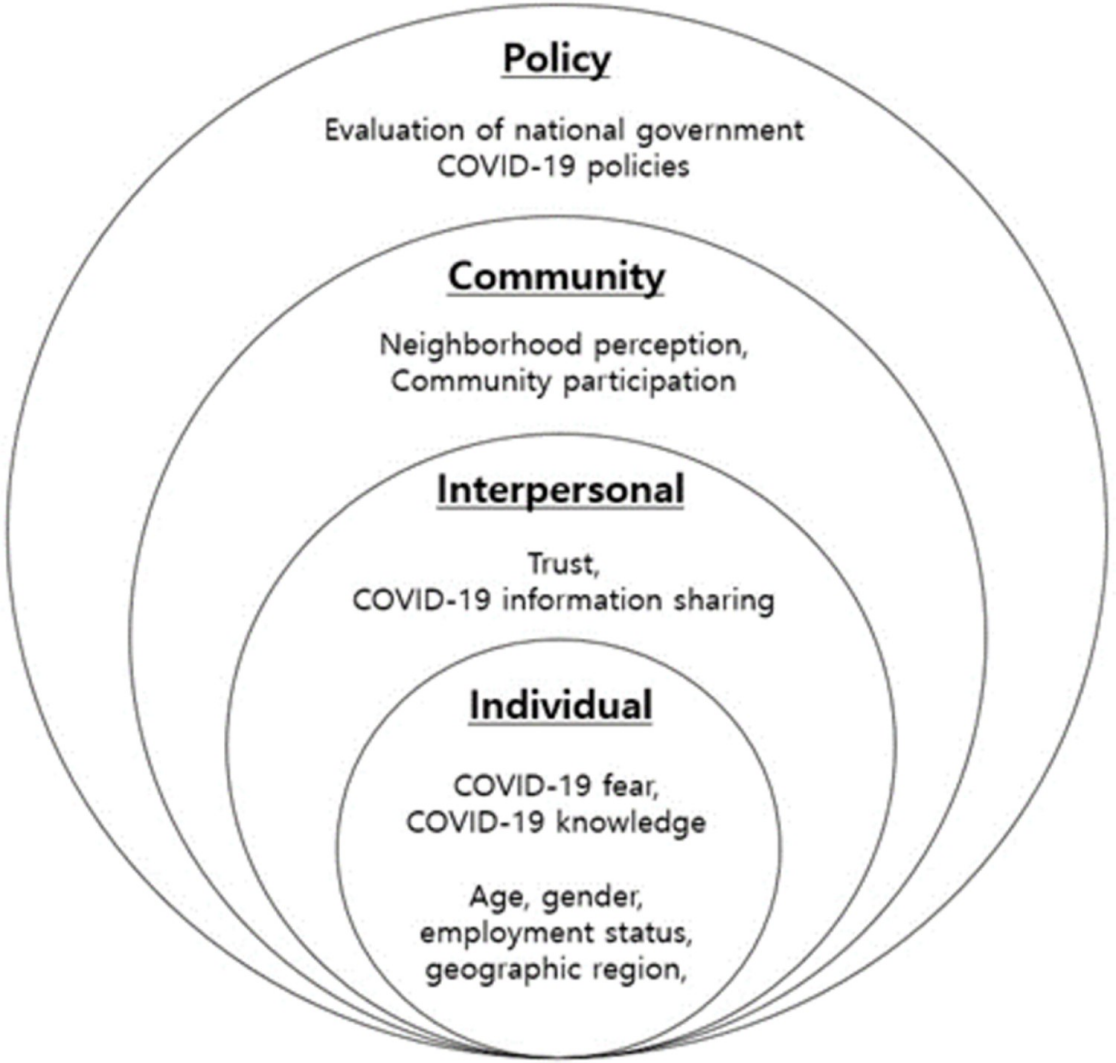
Social-ecological model for COVID-19 preventive behaviors.

By applying the social-ecological model, independent variables at multiple levels were used (Fig 1). The individual-level variables included fear and knowledge of COVID-19, which were adapted from previous studies that developed and validated the measures [10, 35–37].

The fear of COVID-19 was assessed using seven items scored on a 5-point scale (ranging from 1 to 5): 1) “I am afraid of contracting COVID-19”; 2) “I do not feel comfortable when I think about COVID-19”; 3) “Thinking about COVID-19 makes my hands sweat”; 4) “I am worried that COVID-19 will kill me”; 5) “When I hear news about COVID-19 through the media or various media, I feel anxious”; 6) “I am worried about COVID-19, so I cannot sleep”; and 7) “My heart beats fast when I think about COVID-19.” We used the average points (1 = totally disagree; 5 = totally agree) from the seven responses (Cronbach’s alpha [α] = 0.8808). The mean score of all seven items was 3.12 out of 5 (SD = 0.78), suggesting that the participants felt some COVID-19 fear.

The correct knowledge of COVID-19 was measured using the following nine items: 1) “The main clinical symptoms of COVID-19 are fever, coughing, and loss of post-taste”; 2) “Unlike the common cold, nasal congestion, a runny nose, and sneezing are less common in persons infected with COVID-19”; 3) “Currently, there is no cure for COVID-19”; 4) “Eating or contact with wild animals would result in infection with the COVID-19 virus.”; 5) “Eating kimchi prevents COVID-19”; 6) “The COVID-19 virus is airborne”; 7) “COVID-19 is transmitted through the respiratory droplets”; 8) “COVID-19 spreads from human to human”; and 9) “Not all persons with COVID-19 will develop severe symptoms.” The possible answers were “true,” “false,” and “I don’t know.” The correct knowledge of COVID-19 was recoded as follows: Each possible answer was recoded as 1 if the survey participant’s answer was correct and 0 if the answer was incorrect or if the participant answered “I don’t know.” The mean value was used, and the Cronbach’s alpha of the scale was 0.7325.

#### Interpersonal level

Factors at the interpersonal level included interpersonal trust and COVID-19 information shared with others. Interpersonal trust was measured by the trust (1 = not trust at all; 5 = trust completely) of the following six groups: 1) family members, 2) neighbors, 3) acquaintances (friends, coworkers), 4) strangers, 5) foreigners, and 6) religious leaders (e.g., pastors, priests, monks). The average points from the six responses were used, and the Cronbach’s α was 0.7518.

Additionally, COVID-19 information sharing was assessed via two items: whether the participants shared COVID-19 related information with their 1) family members and 2) friends/coworkers (1 = not at all; 5 = totally). The average of the 5-point scale was used, and the Cronbach’s α was 0.7597.

#### Community level

Factors at the community level include neighborhood perceptions and community participation. First, neighborhood perception was assessed via the following four statements: 1) “People in my neighborhood know each other well”; 2) “We often talk about what happens in the neighborhood”; 3) “The neighbors help each other in case of difficulties”; and 4) “The neighbors actively participate in various events and gatherings in the neighborhood.” The responses were also rated on a 5-point Likert scale, ranging from “totally disagree” (1 point) to “totally agree” (5 points). The average of all responses was used, and the Cronbach’s α was 0.9082.

Second, community participation was measured by the survey participants’ engagement and activity in different communities as follows: 1) political parties; 2) labor union organizations/civic organizations/professional unions; 3) religious organizations; 4) clubs (sports, leisure, culture, etc.); 5) civic groups; 6) public gatherings in the local community (e.g., resident organizations); 7) alumni associations; 8) volunteer or donation organizations; and 9) social/economic organizations (e.g., social enterprises). A 5-point Likert scale was used, ranging from “never affiliated” (1 point) to “affiliated and actively participate” (5 points). The average of all responses was used, and the Cronbach’s α was 0.8455.

#### Policy level

To assess the evaluation of national government COVID-19 policies at the policy level, the following eight statements were used: 1) “Anyone can easily access and check the COVID-19 information”; 2) “COVID-19 information is being transparently disclosed to all citizens”; 3) “The government guarantees the participation of the people in deciding the COVID-19 policy”; 4) “The government is collecting opinions from the public in pursuing the COVID-19 policy”; 5) “Government policies are effectively implemented to ensure that people continue to live a stable life in the midst of the COVID-19 crisis”; 6) “The COVID-19 policy is being implemented to protect the health of all citizens”; 7) “The government’s COVID-19 policy is benefiting all the people”; and 8) “The government’s emergency support for those vulnerable to COVID-19 is well underway.” Responses were rated on a 5-point Likert scale, ranging from “totally disagree” (1 point) to “totally agree” (5 points). The average of all responses was used, and the Cronbach’s α was 0.9373.

#### Control variables

Control variables included age group (<30s, 30s, 40s, 50s, 60s), gender (male vs. female), marital status (unmarried, married without children, married with child/children), educational attainment (high school or less, college graduates, graduate school), employment status (unemployed vs. employed), and household income.

### Analysis

A Pearson correlation matrix was used to examine the interrelationships among multilevel factors. Multiple linear regression was used to examine the association between social-ecological predictors and COVID-19 preventive behaviors. A hierarchical model was used in this study. In the first step, individual-level factors (COVID-19 fear and correct knowledge of COVID-19) were fitted. The second model was fitted with interpersonal-level factors (interpersonal trust and COVID-19 information sharing), in addition to the variables in the first model. The third model was built with community-level factors (neighborhood perception and community participation) and variables in the second model. In the fourth model, the policy-level factor (evaluation of national government COVID-19 policies) was included with the variables in the third model. In the final model, socio-demographic variables (age, gender, marital status, educational attainment, and household income) were included, similar to the variables in the previous model. All analyses were conducted using Stata 15.0 software, and the significance level was set at p < .05.

## Results

Overall, the participants reported a high score for preventive behaviors for each measure. With 10 as the full point, the average points of practice were the highest when wearing a mask (mean = 8.85; standard deviation [SD] = 1.93), followed by washing hands after going out (mean = 8.73; SD = 1.75), covering the mouth when coughing and sneezing (mean = 8.64; SD = 1.76), social distancing (mean = 8.25; SD = 1.81), and washing hands before meals (mean = 8.16; SD = 1.98). The overall mean value of all five preventive behaviors was 8.52 (SD = 1.54).

Table 1 presents participants’ characteristics. The mean score for fear of COVID-19 was 3.12 out of 5 (SD = 0.78). Their COVID-19-related knowledge was approximately 56% correct. The mean of interpersonal trust was 2.94/5 (SD = 0.59), which was slightly above the neutral level of interpersonal trust. Compared to trust, they actively shared COVID-19 information with their families and friends (mean = 3.81/5; SD = 0.77). The descriptive statistics of community-level factors revealed that the participants had a neutral perception of their neighborhood (mean = 2.41/5; SD = 0.90), and most were not affiliated with or did not participate in organizations (mean = 1.68/5; SD = 0.72). Notably, the participants had a slightly positive evaluation of COVID-19 policies (mean = 3.46/5; SD = 0.89).

**Table 1:** Characteristics of the survey participants (N = 1,500).

Independent Variables at Multiple Levels	Mean (SD)
***Individual level***	
**COVID-19 fear**	3.12/5 (0.78)
**COVID-19 correct knowledge**	0.56/1 (0.22)
***Interpersonal level***	
**Interpersonal trust**	2.94/5 (0.59)
**COVID-19 information sharing**	3.81/5 (0.77)
***Community level***	
**Neighborhood perception**	2.41/5 (0.90)
**Community participation**	1.68/5 (0.72)
***Policy level***	
**Evaluation of government COVID-19 policies**	3.46/5 (0.89)
**Control Variables**	N (%)
***Socio-demographic characteristics***	
**Age**	
<30	292 (19.47)
30s	273 (18.20)
40s	328 (21.87)
50s	344 (22.93)
60s	263 (17.53)
**Sex**	
Male	760 (50.67)
Female	740 (49.33)
**Marital status**	
Unmarried	620 (41.33)
Married without children	373 (24.87)
Married with children	507 (33.80)
**Educational attainment**	
High school graduates or less	274 (18.27)
College graduates	1,093 (72.87)
Graduate school	133 (8.86)
**Employment status**	
Unemployed	410 (27.33)
Employed	1,090 (72.67)
**Household income**	
Less than $40,000	657 (43.80)
$40,000 or more	843 (56.20)

SD = standard deviation; Note: ₩1 (Korean won) was calculated as approximately $1 for household income.

Among the 1,500 participants, about one-fifth were in their 30s, 40s, 50s, and 60s each, and about half were female. While 41.33% of the participants were unmarried, one-quarter were married with no children, and 33.8% were married with one or more children. Regarding educational attainment, most of the respondents were college graduates (72.87%) or had attended graduate school (8.86%). Finally, most of them (72.67%) were employed, and more than half (56.2%) had a household income of approximately $40,000 or more.

As Table 2 shows, in general, weak correlations were observed among the multilevel factors. The relationship between interpersonal trust and neighborhood perception was the strongest, followed by the relationship between neighborhood perception and community participation, and the relationship between interpersonal trust and community participation. Correct knowledge of COVID-19 had a negative correlation with neighborhood perception and community participation.

**Table 2:** Correlation matrix for multilevel factors (N = 1,500).

	1	2	3	4	5	6	7
**1. Fear**	1.000						
**2. Correct knowledge of COVID-19**	-0.0114	1.000					
**3. Interpersonal trust**	0.0409	**0.1003**	1.000				
**4. COVID-19 information sharing**	**0.1376**	**0.2009**	**0.2789**	1.000			
**5. Neighborhood perception**	**0.2304**	**-0.1034**	**0.4717**	**0.1594**	1.000		
**6. Community participation**	**0.1597**	**-0.0557**	**0.3306**	**0.1207**	**0.3996**	1.000	
**7. Evaluation**	**0.1110**	**0.1298**	**0.2102**	**0.2492**	**0.1618**	**0.0689**	1.000
**of national government COVID-19 policies**							

Numbers in bold are statistically significant at p < .05.

Table 3 shows the social-ecological factors associated with overall COVID-19 preventive behaviors, including all preventive measures. Model 1 includes factors at the individual level, such as COVID-19 fear and correct knowledge. Participants with a higher degree of COVID-19 fear and correct COVID-19 knowledge were more likely to practice preventive measures. The impact of correct knowledge of COVID-19 was stronger than that of fear about COVID-19 (beta = 0.378 vs. 0.144, respectively). In fact, across all models, correct knowledge of COVID-19 was found to be the most influential factor for predicting preventive behaviors. Model 2 shows that, in addition to these individual-level factors, COVID-19 information sharing at the interpersonal level was positively related to preventive behaviors (beta = 0.136), while interpersonal trust was not. Factors at the community level—neighborhood perception and community participation—were negatively associated with COVID-19 preventive behaviors (Model 3), and the impact was stronger for neighborhood perception than community participation (beta = -0.128 vs. -0.086, respectively). At the policy level, a positive evaluation of the national government COVID-19 policies was positively related to participants’ COVID-19 preventive behaviors (Model 4). While all multilevel factors were associated with preventive behaviors when control variables (socio-demographic factors) were included in Model 5, participants who were in their 40s or older, female, and had completed graduate school tended to practice COVID-19 preventive measures more than their younger, male, and less-educated counterparts.

**Table 3:** Social-ecological factors predicting overall COVID-19 preventive behaviors (N = 1,500).

	Model 1	Model 2	Model 3	Model 4	Model 5
**Individual level**					
COVID-19 fear	0.144***	0.101***	0.127***	0.122***	0.117***
	(0.046)	(0.048)	(0.048)	(0.048)	(0.048)
Correct knowledge of COVID-19	0.378***	0.368***	0.341***	0.331***	0.309***
	(0.166)	(0.164)	(0.165)	(0.166)	(0.165)
**Interpersonal level**					
Interpersonal trust		0.041	0.115***	0.104***	0.075**
		(0.108)	(0.071)	(0.071)	(0.072)
COVID-19 information sharing		0.136***	0.184***	0.169***	0.150***
		(0.321)	(0.063)	(0.064)	(0.064)
**Community level**					
Neighborhood perception			-0.125***	-0.128***	-0.117***
			(0.050)	(0.050)	(0.049)
Community participation			-0.089**	-0.086**	-0.089**
			(0.054)	(0.054)	(0.055)
**Policy level**					
Evaluation of national government COVID-19 policies				0.086***	0.089***
				(0.041)	(0.041)
**Socio-demographic**					
**Age** (ref: < 30s)					
30s					0.047
					(0.120)
40s					0.094**
					(0.126)
50s					0.132***
					(0.127)
60s					0.147***
					(0.141)
**Gender** (ref: male)					
Female					0.104***
					(0.074)
**Marital status** (ref: unmarried)					
Married without children					0.000
					(0.112)
Married with child/children					-0.017
					(0.099)
**Educational attainment** (ref: high school graduates or less)					
College graduates					0.052
					(0.099)
Graduate school					0.090**
					(0.151)
**Employment** (ref: unemployed)					
Employed					-0.042
					(0.086)
**Household income**					0.020
					(0.016)
Adjusted R^2^	0.1620	0.1832	0.2029	0.2091	0.2371

All standardized regression coefficients (Beta)

Standard errors (SE) in parentheses

*** p < .001

** p < .01

* p < .05

Considering the adjusted R-squared values of each model, the individual-level factors and interpersonal factors accounted for the most predictability, whereas community-level and policy-level factors explained only a small portion of predictability. Nonetheless, the adjusted R-square of Model 5 suggests that factors at each level contribute to the predictability of COVID-19 preventive behavior.

## Discussion

As previously acknowledged, Korea might have been able to efficiently control COVID-19 because of the support from its citizens in addition to its governmental efforts, and all the participants indicated that they were actively engaged in preventive practices. In line with the findings of previous studies regarding the applicability of the social-ecological model to various issues [8, 13, 32–34], the current study found that the factors at each level of the social-ecological model predicted an individual’s COVID-19 preventive behaviors during the pandemic in Korea, suggesting that multilevel efforts are needed to promote preventive behaviors.

Compared to the factors at the upper levels, factors at the individual and interpersonal levels were more suited to explain individuals’ practice of COVID-19 preventive measures, which suggests that there need to be more policies targeting these lower levels. For example, correct knowledge of COVID-19 is positively associated with preventive behaviors, suggesting that more support is needed to disseminate correct information and resist COVID-19 misinformation. This is because previous studies have also confirmed that individuals with correct information on COVID-19 are more likely to practice preventive measures [38, 39].

Additionally, as this study found that COVID-19 preventive behaviors differ by demographic characteristics at the individual level, a theory-based tailored intervention targeting younger males with easily understandable information should be developed and disseminated. It may be necessary for clinicians to consider developing, disseminating, and implementing place-based interventions (e.g., worksite-based interventions for the employed and university-based interventions for young adults who are less likely to practice preventive measures). The wellness center on campus could be an ideal place for clinicians to disseminate interventions for young adults.

This study contributes to the theory and the expansion of the existing, limited literature on COVID-19 preventive behaviors by applying the social-ecological model and suggests that other multilevel factors beyond the individual level could have an impact on an individual’s health behavior during the COVID-19 pandemic. Based on the impact of multilevel factors on preventive behavior, there are multilevel policy implications based on the findings of the current study. First, at the interpersonal level, sharing COVID-19 information with family members, friends, and coworkers could positively impact individuals’ preventive behaviors. Therefore, more efforts should be made to fight against COVID-19 misinformation and to disseminate correct information. Moreover, COVID-19 information should be provided to individuals who lack a social network or have a narrow one, including those who live alone and are thus isolated from receiving information. Additionally, interpersonal trust was positively related to preventive behavior after adding higher-level factors and controlling for socio-demographic characteristics. Thus, more efforts should be made to further increase interpersonal trust. As institutional trust could promote interpersonal trust [40], the government could make more efforts to increase institutional trust through transparent information disclosure and communication.

Second, at the community level, a more positive perception of one’s neighborhood and a higher degree of participation in communities were found to be negatively related to individuals’ COVID-19 preventive behaviors. Considering these factors as broadly defined social capital at the community level, the findings of the current study contradict the findings of earlier studies that a higher degree of social capital has a positive association with preventive health behaviors [17] and a negative association with mortality and mobility [41], focusing on most Western countries, including the United States and European countries. One possible explanation for this negative relationship between community-level factors (neighborhood perception and community participation) and preventive behaviors could be that individuals who trust their neighbors and the members of their affiliated organizations expect them to practice COVID-19 preventive measures more attentively. This might negatively impact individuals’ diligent practice of preventive behaviors, which is analogous to the previous study [42], which found that a country with a higher level of trust tends to have more COVID-19 deaths. However, this explanation contradicts an earlier study [34], which found a positive relationship between perceived adherence to others’ preventive behaviors and self-adherence with the control of collectivism, such as collective responsibility, collective efficacy, and empathy. To precisely examine the relationship between community-level factors and preventive behaviors in Korea, trust and collectivism at the community level should be further considered in future studies. Future studies also need to control the characteristics of the neighborhood or community (e.g., the number of COVID-19 confirmed cases, deaths, and socioeconomic status). Moreover, as previous studies have indicated [13, 14], the motivation for preventive behaviors (self-interested vs. prosocial) might impact preventive behaviors. In other words, community-level factors might be positively associated with an individual’s preventive behaviors if the individual has prosocial motivation rather than self-interested motivations. Jordan et al. [14] pointed out the importance of distinguishing between the motivations of “don’t get it” or “don’t spread it”; future studies need to similarly consider the motivations.

Finally, at the policy level, the government should try to implement better and more transparent policies, as the current study found a positive relationship between the evaluation of governmental policy and preventive behaviors. According to a previous study [8] that compared 10 different countries, trust in government was significantly related to risk perception only in Korea and Spain. As this study also found that risk perception was related to preventive behaviors, efforts to increase trust in government by disclosing transparent communication and policy processes could be helpful in the practice of preventive behaviors. For example, the Korea Disease Control and Prevention Agency (KCDA) website provides COVID-19-related statistics, corrects misinformation about COVID-19, and promotes preventive practices, emphasizing the importance of wearing a mask and social distancing. However, the website is a one-way medium of communication, as there is no way for Korean citizens to ask questions or provide comments.

The current study has several limitations. First, it excluded children and adolescents younger than 19 years and older adults over 70 years old. As earlier studies have confirmed disparities in COVID-19 preventive practices by age [9, 37], future studies should include a wider age range of the participants. Second, all preventive practices were self-reported. Considering the stigma against COVID-19 [43], the participants might have been more generous in reporting their practices. Third, because of data limitations, this study did not control for individuals’ current health status. As people with chronic diseases are more vulnerable to COVID-19 [44, 45], they might be more cautious and thus practice preventive measures better. In addition to the current health status, this study did not control whether the participants were affected by COVID-19. In line with the findings of a previous study [25], it was found that a COVID-19 infection could influence individuals’ preventive behaviors. Finally, although the current study examined multilevel factors associated with various kinds of COVID-19 preventive behaviors, getting a COVID-19 vaccination, as recently recommended by the WHO [46], was not included because the data were collected when the COVID-19 vaccination had not yet been introduced in Korea. As concerns about COVID-19 vaccination safety and vaccine hesitancy are on the rise among people [47, 48], future studies could compare the multilevel factors associated with vaccination with other preventive measures.

Despite these limitations, the current study is one of the first attempts to understand individuals’ preventive behaviors in Korea by applying a multilevel framework. Starting from the current study, future studies could examine the multilevel impacts on preventive behaviors across different countries. Recently, a new phenomenon called “vaccine tourism,” which involves traveling across states or even abroad to get a COVID-19 vaccination—a type of preventive behavior—has emerged [49, 50]. Since the COVID-19 pandemic is a global phenomenon with transnational movement among individuals, factors at the transnational level (e.g., transnational regulations, quarantine rules) beyond the national policy level, which might impact preventive behaviors, should be further examined.
